# University of Louisville

**Published:** 1847-12

**Authors:** 


UNIVERSITY OF LOUISVILLE.

The classes in both the Law and Medical Department of the University of Louisville are larger than they were last winter. The annual circular, to be issued the first of January, will exhibit remarkable prosperity in the institution. We may say. in one word, that the spacious rooms in the Medical College are full.

